# Detection of Tumor DNA in Bronchoscopic Fluids in Peripheral NSCLC: A Proof-of-Concept Study

**DOI:** 10.1016/j.jtocrr.2023.100596

**Published:** 2023-10-19

**Authors:** Gwenaëlle Arhant, Samy Lachkar, Pierre-Alain Thiebaut, Florent Marguet, Aude Lamy, Luc Thiberville, Mathieu Salaün, Florian Guisier, Jean-Christophe Sabourin, Nicolas Piton

**Affiliations:** a Department of Pathology, Normandie Univ, UNIROUEN, Institut national de la santé et de la recherche médicale (INSERM) U1245, CHU Rouen, Rouen, France; b Department of Pneumology, CHU Rouen, Rouen, France; c Department of Pneumology, Normandie Univ, UNIROUEN, LITIS Lab QuantIF team EA4108, CHU Rouen, and Institut national de la santé et de la recherche médicale (INSERM) CIC-CRB 1404, Rouen, France

**Keywords:** Liquid biopsy, Tumor genotyping, Non–small cell lung carcinoma, Peripheral lung nodule, Radial-EndoBronchial UltraSound, Targeted therapy

## Abstract

**Introduction:**

DNA genotyping from plasma is a useful tool for molecular characterization of NSCLC. Nevertheless, the false-negative rate justifies the development of methods with higher sensitivity, especially in difficult-to-reach peripheral lung tumors.

**Methods:**

We aimed at comparing molecular analysis from the supernatant of guide sheath flush fluid collected during radial-EndoBronchial UltraSound (r-EBUS) bronchoscopy with plasma sampling and tumor biopsies in patients with peripheral NSCLC. The DNA was genotyped using high-throughput sequencing or the COBAS mutation test. There were 65 patients with peripheral lung tumors subjected to concomitant sampling of guide sheath flush supernatant, plasma tumor DNA, and tumor biopsy and cytology using r-EBUS. There were 33 patients (including 24 newly diagnosed with having NSCLC) with an identifiable tumor mutation in the primary lesion selected for the comparative analysis.

**Results:**

Guide sheath flush-based genotyping yielded a mutation detection rate of 61.8% (17 of 24 mutated *EGFR*, one of two *ERBB2*, one of one *KRAS*, one of one *MAP2K*, one of four *MET*, and zero of one *STK11*), compared with 33% in plasma-based genotyping (*p* = 0.0151). Furthermore, in eight of 34 r-EBUS without tumor cells on microscopic examination, we were able to detect the mutation in four paired guide sheath flush supernatant, compared with only two in paired plasma.

**Conclusion:**

The detection of tumor DNA in the supernatant of guide sheath flush fluid collected during r-EBUS bronchoscopy represents a sensitive and complementary method for genotyping NSCLC.

## Introduction

Circulating tumor DNA (ctDNA) is the fraction of cell-free DNA (cfDNA) derived from tumors that can be found in various body fluids. The genotyping of ctDNA is routinely used in NSCLC at the time of diagnosis to detect oncogenic alterations or during the first or second generation of EGFR tyrosine kinase inhibitor treatment to detect resistance mutations.^1^ With the advent of targeted therapies and immunotherapy, multiple and sequential strategies have been developed to treat lung cancer, leading to consecutive molecular testing.^2^ In some cases, ctDNA analysis makes it possible to invasive procedures in patients with typically poor general condition or advanced disease at diagnosis, thus contraindicating surgery.^3^ In addition, ctDNA analysis can preserve valuable material obtained from biopsy and cytology for pathologic analysis, which may be insufficient for molecular characterization.^4^ Furthermore, tissue samples collected during bronchoscopy can be very small, as a result of minimally invasive techniques such as radial-EndoBronchial UltraSound (r-EBUS)–assisted biopsy with a guide sheath (GS). Unfortunately, in approximately 30% of cases, these samples are noncontributory for genomic analysis owing to a lack of available tumor tissue.^5^, ^6^, ^7^ In this context, ctDNA analysis could represent an alternative for genomic characterization.

Other body fluids have been screened for NSCLC diagnosis, such as pleural effusion,^8^^,^^9^ urine,^10^^,^^11^ cerebrospinal fluid,^12^ saliva,^13^ sputum,^14^ and even expired air.^15^ Cell-free fluids such as supernatant collected during fine-needle biopsy procedures have also been successfully used for genomic profiling.^16^^,^^17^

Given the poor sensitivity of genotyping cfDNA from the blood, we hypothesized that the quantity and quality of tumor DNA would be more adequate in liquid samples obtained in direct proximity to the tumor during bronchoscopy. Moreover, molecular analysis of bronchoscopic fluid could be used to preserve tumor tissue for histologic and immunohistologic characterization. The aims of this proof-of-concept study were to genotype liquid biopsy specimen, collected during bronchoscopy, in the proximal environment of bronchial tumors, and to compare the molecular profiles with those obtained from the tumor biopsy and routine blood samples.

## Materials and Methods

This monocentric study was conducted from November 2015 to July 2019. During this period, patients with peripheral lung nodules suspicious of lung cancer were routinely subjected to r-EBUS with the GS technique for diagnosis as described elsewhere.^5^^,^^6^

### r-EBUS Procedure and Sampling

**Figure 1:**
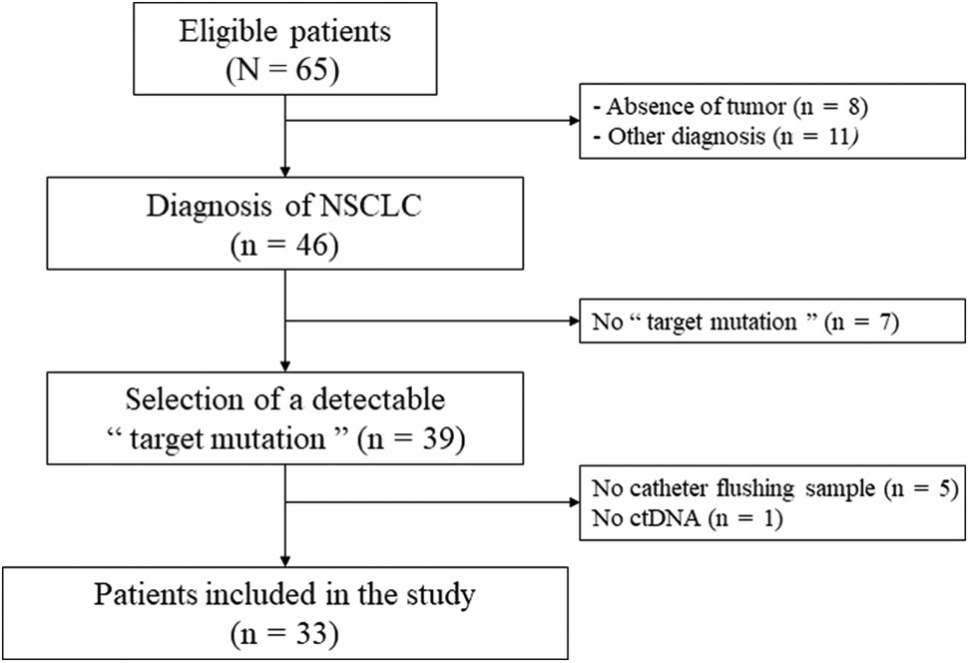
Flowchart of patient selection. Patients with highly suspected or confirmed NSCLC with a detectable “target” mutation were eligible. Selection of “target mutation” was extracted from tissue sample genotyping. ctDNA, circulating tumor DNA; GSF-s, supernatant of guide sheath flush fluid.

The routine procedure included distal echo-guided biopsies (four to six samples), brushing in the tumor through the catheter kit using the dedicated microforceps and microbrush (Olympus, K101), and flushing of the catheter using 5 mL of sterile saline. A 5-mL blood sample was also taken before the bronchoscopy procedure for ctDNA analysis. All patients with confirmed peripheral lung NSCLC who had the complete procedure, including plasma and GS flush sampling, were selected for this retrospective study (Fig. 1). From these patients, those harboring a “target” mutation from the primary tumor in *EGFR*, *KRAS*, *HER2*, *MET*, *MAP2K*, or *STK11* genes were included. In case of co-occurrences of somatic mutations, the mutation with a higher allelic ratio was chosen as the “target.”

### Liquid Biopsy

During the bronchoscopy procedure, paired samples were collected from each patient. One sample was the plasma, which was collected in ethylenediaminetetraacetic acid collection tubes at the time of the bronchoscopy procedure. The other sample was the supernatant of GS flush (GSF-s), which was collected immediately after the tissue biopsy. In addition to these samples, whenever possible, bronchoalveolar lavage (BAL) was also collected just before the tissue biopsy. This involved the use of 50 mL of physiological serum to collect the BAL sample.

All samples were transmitted immediately to the pathology department or within 4 hours with storage at 4°C. On collection, all samples including the blood, BAL, and GSF-s were promptly transferred to 20 mL Falcon tubes and subjected to a preliminary centrifugation at ×700 at 4°C for 10 minutes. After the centrifugation, the cell pellet obtained from GSF was transferred to the cytology laboratory for standard analysis. Meanwhile, the supernatant obtained from the first centrifugation was subjected to a second centrifugation step at ×2700 at 4°C for 10 minutes, after which the cell-free supernatant was cryopreserved at −80°C for future use.

### DNA Extraction

For DNA extraction, the QIAamp circulating nucleic acid kit (Qiagen, Hilden, Germany) was used, following the manufacturer's instructions. One plasma sample was extracted using the COBAS extraction kit without cfDNA quantification (which accounts for the absence of data for paired sample number 15).

### Genotyping

For DNA extraction, the QIAampR circulating nucleic acid kit (Qiagen, Hilden, Germany) was used following the manufacturer’s instructions. One plasma sample was extracted using the COBAS extraction kit (Roche, Rotkreuz, Switzerland) without cfDNA dosage, which explains the missing data for paired sample number 15 in Table 1.

**Table 1:** Results of Paired GSF-s, Plasma and Histology or Cytology Samples

Sample Number	GSF-s Mutation Detection	Plasma Mutation Detection	GSF-s cfDNA Concentration	Plasma cfDNA Concentration	GSF-s Mutation Allelic Ratio	Plasma Mutation Allelic Ratio	Tumor Cells on Microscopic Examination
1	-	-	4.36	30.4	NA	NA	+
2	+	+	120	70.1	33.1	15.6	+
3	+	+	0.2	0.39	40.5	9.7	-
4	+	+	7.2	71.2	91.1	4.48	+
5	+	+	0.3	9.16	39.6	15.1	+
6	+	-	15.93	0.11	11.7	NA	+
7	+	+	9.04	0.76	24.8	1.3	+
8	+	-	170	13.7	2.37	NA	**+**
9	-	-	19.4	26.4	NA	NA	+
10	+	+	165	31.2	66.06	28.9	+
11	+	-	10.5	1.09	2.6	NA	+
12	+	-	191	1.28	11.2	NA	**+**
13	+	-	14.6	0.27	57.5	NA	+
14	+	-	0.38	0.9	6.1	NA	-
15	+	+^a^	4.81	Missing	NA	NA	-
16	-	-	37.5	1.8	NA	NA	+
17	-	-	0.44	3.14	NA	NA	+
18	-	-	0.75	9.51	NA	NA	+
19	+	-	1.48	0.37	26.8	NA	+
20	-	-a	57.1	75.2	NA	NA	+
21	+	-	20.5	78	13.9	NA	**+**
22	-	-	12.4	2.04	NA	NA	-
23	+	+	22.2	6.33	NA	40.5	+
24	-a	-	1.53	0.62	NA	NA	-
25	+	+	6.78	0.76	25.2	1.3	+
26	+	+	0.96	1.23	15.2	2.7	+
27	-	-	10.4	0.99	NA	NA	+
28	-	-	3.13	1.12	NA	NA	+
29	+	+	46.7	16.3	66.6	66.6	+
30	-	-	0.27	0.58	NA	NA	-
31	+	-	3.69	0.373	46.9	NA	**+**
32	-	-	13.2	4.28	NA	NA	-
33	-a	-	14.6	3.67	NA	NA	+
34	+	-	5.56	7.19	NA	NA	-

*Note:* A total of 34 Sets of samples in 33 patients are reported (one patient with two sets of samples).

-, negative; +, positive; cfDNA, cell-free DNA; GSF-s, guide sheath flush supernatant; NA, not applicable.

a Indicates samples for which COBAS *EGFR* mutation test was used without allelic ratio data available (GSF-s cfDNA concentration expressed in ng/μL, allelic ratio expressed in %).

Extracted DNA was analyzed using either high-throughput sequencing techniques or COBAS technology, depending on the period of time of the molecular analysis in our laboratory. Two different high-throughput sequencing techniques were used. The first technique was based on the Tumor Hotspot MASTR Plus kit (Multiplicom, Agilent Technologies, Santa Clara, CA) for library creation and the MiSeq sequencer (Illumina). Data were aligned using BWA-GATK-07.6a-3.1.1 software on the hg19 version of the human genome. The second technique, using Unique Molecular Identifiers technology, was based on the VariantPlex solution (Archer) for library creation. Data were aligned using Archer Analysis 6.2.7 software. Variant calling was performed with VarScan2, and data were annotated using Alamut software (Sophia Genetics, Boston, MA). The covered regions are annotated in Supplementary Annex 1.

COBAS technology is a real-time polymerase chain reaction technique performed with the COBAS EGFR mutation test version 2 kit. The tested *EGFR* mutations are detailed in Supplementary Annex 2.

### Statistics

Data were analyzed using Prism 8.0.2 (263) software (GraphPad, Insight Venture Partners, New York, NY). Binary and discrete variables are presented as numbers and percentages, with a 95% confidence interval (CI). Quantitative variables are presented as means for normally distributed data (verified using the Kolmogorov-Smirnov test) or as medians with interquartile range (Q1–Q3) for non-normally distributed data. The independence between two qualitative variables was tested using the chi-square test, whereas the independence between the quantitative variables was tested using the Mann-Whitney nonparametric test. All statistical tests were two tailed, with *p* values considered significant if below 0.05.

### Ethics

Ethical approval was obtained from the local ethics committee (agreement number E2022-28) and according to the agreement of the tumor biobank of Rouen University Hospital (tissue sample collection number DC2008-689) by the institutional review board of Rouen University Hospital and by the French Ministry of Scientific Research. Oral informed consent for the procedure was obtained for all patients. According to the ethics committee, a written informed consent was not required, as the research did not modify the usual bronchoscopic procedure.

## Results

### Cohort Description

**Figure 2:**
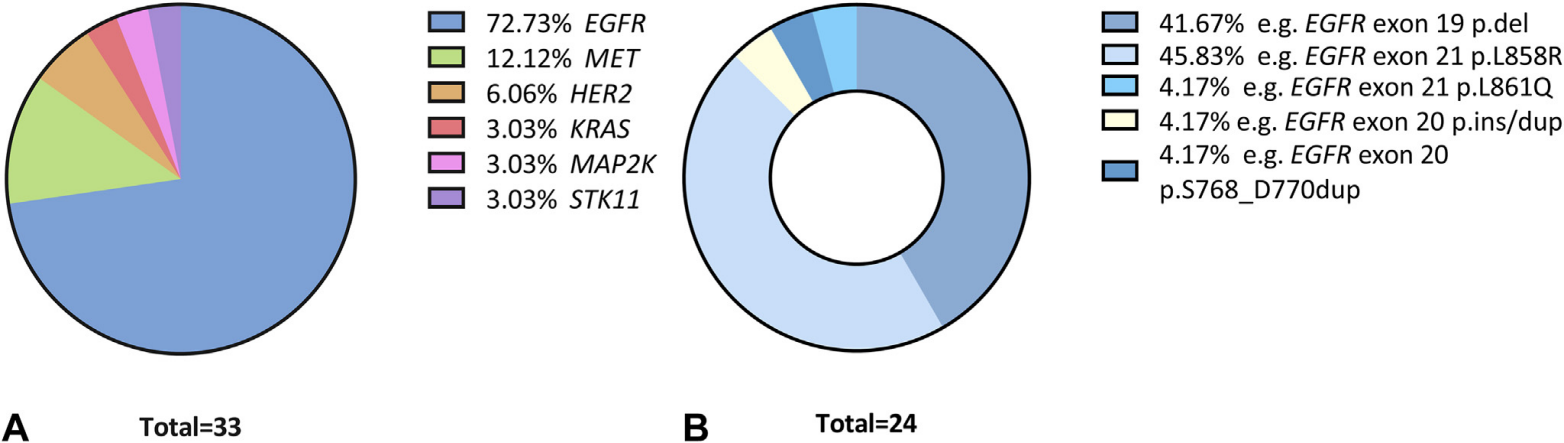
Distribution of mutated genes (*A*) detected in the 33 tumors (*B*) with a focus on the distribution of mutations of *EGFR* only. del, deletion; dup, duplication; ins, insertion.

A total of 65 patients were eligible for the study, from which 33 were included in the study based on the availability of samples and the detection of a “target” mutation from the biopsy, the brush, or another tumor sampling in case of negative r-EBUS result (Fig. 1). Most patients were never smokers (n = 22, 67%) and had stage IV disease at the time of collection (n = 24, 73%) (Table 2). From these 33 patients, r-EBUS was performed at the diagnostic stage in 23 and 10 at relapse. A total of 34 paired samples (i.e., blood and supernatant of GSF-s) were collected from our cohort, with one patient having two sets of samples. Mean size of tumors was 3.6 cm (SD = 1.5). BAL performed just before biopsy was available for three patients. Tumor cells were obtained from r-EBUS in 22 of 33 patients (24 samples), including 18 from biopsy and four from the brush only. Among the 12 patients with negative r-EBUS result, the diagnosis was obtained from a previous sampling in four cases, from a further r-EBUS procedure in four patients, a surgical resection in three patients, and a computed tomography-guided transthoracic biopsy in one patient. The detected mutations are presented in Fig. 2*A* and mostly consisted of *EGFR* mutations (Fig. 2*B*).

**Table 2:** Patient and Tumor Characteristics at Bronchoscopy

Category	Characteristics	n (%)	Type of EGFR mutations
Sex			
	Female	28 (85)	
	Male	5 (15)	
Age (in y)			
	Mean (SD)	67 (±12)	
	Range	43–89	
Smoking status			
	Current	1 (3)	
	Former	10 (30)	
	Never	22 (67)	
AJCC/UICC stage (eighth edition)			
	I	4 (12)	
	II	1 (3)	
	III	2 (6)	
	IV	24 (73)	
	Missing data	3 (9)	
WHO performance status score			
	0	28 (88)	
	1	3 (9)	
	4	1 (3)	
	Missing data	2 (6)	
Known professional exposure		4 (12)	
Patients treated by surgery		10 (30)	
Stage of disease			
	Initial diagnosis	24 (73)	
	Progression/relapse	9 (27)	
Mutated gene			
	*EGFR*	24 (73)	
			11 L858R (46%)
			10 del19 (42%)
			3 others (L861Q, del20, duplication) (12%)
	*MET*	4 (12)	
	Others (*MAP2K*, *STK11*, *KRAS*)	3 (9)	
	*HER2*	2 (6)	

AJCC, American Joint Committee on Cancer; del 20, deletion exon 20; UICC, Union for International Cancer Control.

### GSF-s Samples Versus Plasma Samples

**Figure 3:**
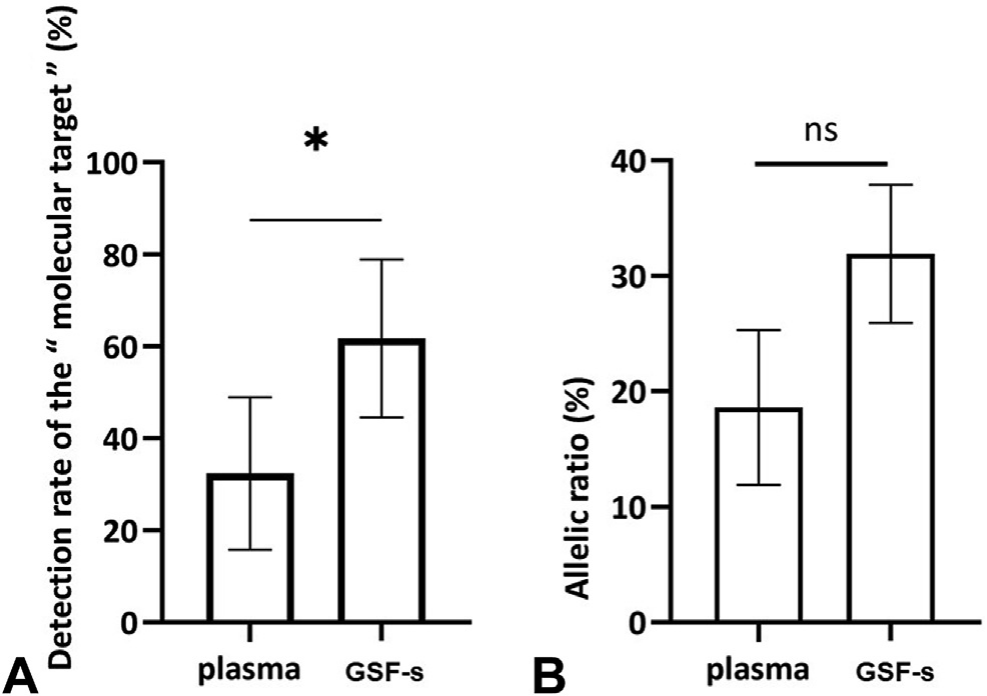
(*A*) Mutation detection rates in the 34 paired GSF-s and plasma samples (whisker plots indicating the mean with 95% CI; *p* = 0.0151). (*B*) Allelic ratio in the 34 GSF-s (n = 21) and plasma (n = 10) samples (whisker plots indicating the median with IQR; *p* = 0.1828). CI, confidence interval; GSF-s, guide sheath flush supernatant; IQR, interquartile range; ns, not significant.

Of the samples, 94% were analyzed using next-generation sequencing (NGS) and 6% using COBAS technology. The mutation detection rate in the GSF-s samples was 61.8% (95% CI: 44.55–78.98), which was significantly higher than that from concomitant plasma samples (32.4%, 95% CI: 15.78–48.92), with a *p* value of 0.0151 (Fig. 3*A*). Furthermore, the allelic ratio tended to be higher in the GSF-s samples than in the plasma samples, with a median of 25% and 12%, respectively, although the difference was not statistically significant (*p* = 0.18) (Fig. 3*B*). Interestingly, 10 of 21 patients (48%) with a mutation detected in the GSF-s samples were negative for plasma analysis (Table 1). Nevertheless, all 11 plasma samples that tested positive were also positive in the GSF-s samples (Table 1).

The rate of technical failures seemed similar for GSF-s and plasma samples, with 11 (32%) and seven (21%), respectively (*p* = 0.27). All technical failures observed in the plasma samples were due to noncovered regions of interest, whereas in the GSF-s samples, five failures were observed during library creation, two samples were not covered by the regions of interest, and two COBAS test results were invalid.

### Diagnostic Efficacy of GSF-s Compared With Tissue Sampling

Among the 34 paired samples analyzed, eight (24%) were negative for tumor cells on pathologic analysis at the time of r-EBUS sampling, including on GSF-s cytologic examination (i.e., no tumor cell was observable) (Table 1). Interestingly, the “target mutation” was detected in four of these eight cases in paired GSF-s, compared with only two in paired plasma. Conversely, among the 24 of 34 samples that were positive for tumor cells on pathologic examination, nine (38%) failed to detect mutation in GSF-s.

In terms of clinical implications, in one patient with previously known NSCLC and *EGFR* mutation, plasma and GSF-s allowed for the detection of an *EGFR* exon 20 p.T790M resistance mutation, despite the absence of tumor cells on pathologic examination. This enabled early treatment with osimertinib (a third-generation tyrosine kinase inhibitor), leading to marked tumor regression.

In addition, two patients had mutation detection only in GSF-s, one at the time of diagnosis and the other at relapse for NSCLC, whereas other samples were noncontributory.

### BAL Results

In three patients, BAL was also performed in close proximity to the observed tumor. Although pathologic examination did not retrieve tumor cells, a target mutation was detected in two BAL-s, which was also detected in GSF-s but only in one paired plasma sample. Of note, paired GSF-s samples presented a higher allelic ratio than corresponding BAL-s.

## Discussion

In this study, we investigated the potential of genotyping “local” liquid biopsies, collected in the proximal environment of peripheral lung tumors, during r-EBUS performed for the diagnosis of NSCLC. To our knowledge, this is the first study to analyze GSF-s material for molecular and diagnosis purposes. Our results reveal a significantly higher likelihood of detecting mutations in GSF-s samples than in paired plasma samples, with a sensitivity of 61.8% compared with 32.4% (*p* = 0.0151). Importantly, four patients had mutation detection only in GSF-s samples despite the absence of tumor cells on biopsy and cytology analyses, which potentially enables early diagnosis or modification of therapeutic line, a critical issue in this aggressive disease.

Furthermore, although performed in only three patients, we found that BAL was sufficient for molecular profiling of tumors, without tissue sampling, for example, in cases of high hemorrhagic risk or if the tumor is not reachable using r-EBUS. Nevertheless, this would require validation in a larger cohort.

In addition to tissue biopsies, cfDNA analysis in the plasma is routinely performed for genomic profiling in NSCLC.^4^^,^^18^ Our work suggests that adding GSF-s genotyping, sampled during the same bronchoscopy, could increase the likelihood of detecting actionable alterations without any additional risk for the patient. Moreover, one of the main limitations of plasma samples is the low allelic ratio owing to the very small amount of ctDNA released in the bloodstream.^1^^,^^18^ Several studies have described the high potential of molecular analyses performed in cell-free supernatants from cytologic samples or catheter rinse in a variety of solid tumors.^19^, ^20^, ^21^, ^22^ Our study is in line with these findings, as we observed that the supernatant from cytologic samples can contain tumor DNA regardless of the presence of tumor cells in the cell fraction.^20^

Overall, our study reveals the potential of GSF-s genotyping for the diagnosis and management of NSCLC, particularly in cases where tissue sampling is not feasible or in case of the absence of tumor cells in biopsy/cytology sampling. Our findings also suggest that BAL alone could be a promising alternative to tissue sampling for molecular profiling of tumors. Further studies are needed to validate these results and to determine the optimal use of GSF-s and BAL in clinical practice.

In our cohort, nine (26%) GSF-s samples failed technical analysis, probably owing to preanalytical parameters. The time interval between blood collection and DNA extraction is a critical factor affecting cfDNA quality,^1^ and the time must be under 2 hours.^23^ In our study, the time between collection and extraction was not systematically measured, and GSF-s samples may have been altered, leading to low DNA quality and explaining the failures of library creation and invalid samples for COBAS analysis. Another hypothesis is that the quality of the DNA collected in the periphery of the tumor is different from that of the plasma, requiring specific preanalytic treatment.

Nonetheless, GSF-s fluid is interesting because it allows a minimally invasive collection of samples from the catheter, without additional bronchoscopic intervention, usually performed in our center for cytologic analysis. Several biases can be noted in our study, mainly regarding the selection of patients, based on clinically and radiologically suspected NSCLC harboring an easily detectable “target” mutation, which was mainly represented by *EGFR* mutations at the time of our study. This selection explains that most patients were women, never smokers, and obviously the predominance of *EGFR* mutations in our cohort. Another major limitation is the use of DNA extraction kits and NGS panels that are originally designed for formalin-fixed, paraffin-embedded tissues which could explain the low detection rate in plasma and GSF-s samples, owing to insufficient sequencing coverage for cfDNA genotyping. Adapted DNA extraction kits and NGS panels, specially designed for liquid samples, could raise the detection rate for GSF-s and plasma samples.

On the basis of our findings, we suggest that liquids collected in the proximity of tumor site (GSF-s or BAL-s) could be suitable for NSCLC genotyping, with a higher yield than paired plasma samples. These procedures are simple and do not induce additional risks for these patients who are frequently in poor condition. Moreover, GSF-s and BAL-s sampling sometimes allow the salvage of defective biopsies, with a higher DNA quality and concentration than in the plasma samples.

## CRediT Authorship Contribution Statement

**Gwenaëlle Arhant**: Formal analysis, Investigation, Data curation, Writing—original draft, Writing—review and editing, Visualization.

**Samy Lachkar**: Conceptualization, Methodology, Validation, Formal analysis, Investigation, Writing—original draft, Writing—review and editing, Visualization.

**Pierre-Alain Thiebaut**: Writing—original draft, Writing—review and editing, Visualization.

**Florent Marguet**: Investigation.

**Aude Lamy**: Methodology, Validation, Formal analysis, Investigation.

**Luc Thiberville**: Conceptualization, Methodology, Validation, Formal analysis, Investigation, Writing—original draft, Writing—review and editing, Visualization.

**Mathieu Salaün**: Conceptualization, Methodology, Investigation.

**Florian Guisier**: Conceptualization, Methodology, Validation, Formal analysis, Investigation, Writing—original draft, Writing—review and editing, Visualization.

**Jean-Christophe Sabourin**: Resources, Funding acquisition.

**Nicolas Piton**: Conceptualization, Methodology, Validation, Formal analysis, Investigation, Resources, Data curation, Writing—original draft, Writing—review and editing, Visualization, Supervision, Project administration, Funding acquisition.

## Permissions

Ethical approval was obtained according to the agreement of the local ethics committee (agreement no. E2022-28) and of the tumor biobank of CHU Rouen (tissue sample collection no. DC2008-689) by the institutional review board of CHU Rouen and the French Ministry of Scientific Research.

## Patient Consent

Oral informed consent for the procedure was obtained for all patients. According to the ethics committee, a written informed consent was not required, as the research did not modify the usual bronchoscopic procedure.

## Supplementary material

### Annex 1

For tumor Hotspot MASTR Plus kit (Multiplicom), the covered regions were *AKT1* (exon 3), *ALK* (exons 20–29), *BRAF* (exons 11–15), *CDKN2A* (exons 1–3), *CTNNB1* (exon 3), *DDR2* (exons 3–18), *EGFR* (exons 18–21), *ERBB2* (exons 19–21), *ERBB4* (exons 10–12), *FGFR2* (exons 7, 10, 12), *FGFR3* (exons 7, 9, 14, and 15), *H3F3A* (exon 2), *HIST1H3B* (exon 1), *HRAS* (exons 2–4), *IDH1* (exon 4), *IDH2* (exon 4), *KIT* (exons 8–11, 13, 14, 17, 18), *KRAS* (exons 2–4), *MAP2K1* (exons 2, 3), *MET* (exons 2, 10, 14–20), *NRAS* (exons 2–4), *PDGFRA* (exons 12, 14, 18), *PIK3CA* (exons 2, 3, 10, 11, 21), *PIK3KR1* (exons 11–13), *PTEN* (exons 1–9), and *STK11* (exons 1–9). The threshold of analytical sensitivity was 5% of allelic ratio.

For the VariantPlex solution (Archer), the covered regions were *AKT1* NM_005163.2: exons 3 and 6; *ALK* NM_004304.5: exons 20–25; *BRAF* NM_004333.6: exons 11 and 15; *CTNNB1* NM_001904.4: exon 3; *DDR2* NM_006182.3: exons 12–18; *EGFR* NM_005228.4: exons 3, 7, 15, 18–21; *EIF1AX* NM_001412.4: exons 1–7; *ERBB2* NM_004448.4: exons 10, 19–21, 24; *ERBB4* NM_005235.3: exons 10 and 12; *FGFR1* NM_015850.3: exons 4, 7, 8, 13, 15, 17; *FGFR2* NM_000141.4: exons 7–9, 11, 12, 14, 16; *FGFR3* NM_000142.4: exons 7–10, 14–16, 18; *GNAS* NM_000516.4: exons 6–9; *H3-3A* (alias H3F3A) NM_002107.7: exon 2; *H3C2* (alias HIST1H3B) NM_003537.4: exon 1; *HRAS* NM_005343.3: exons 2–4; *IDH1* NM_005896.4: exons 3 and 4; *IDH2* NM_002168.4: exon 4; *KIT* NM_000222.2: exons 2, 8–11, 13–15, 17, 18; *KRAS* NM_004985.5: exons 2–5; *MAP2K1* NM_002755.4: exons 2, 3, 4, 6; *MET* NM_000245.4: exons 2, 11, 14–21; *NRAS* NM_002524.5: exons 2–5; *PDGFRA* NM_006206.4: exons 12, 14, 15, 18, 23; *PIK3CA* NM_006218.4: exons 2, 5, 7, 8, 10, 14, 19, 21; *PTEN* NM_000314.8: exons 1–9; *RET* NM_020630.5: exons 10, 11, 13–16; *ROS1* NM_002944.2, exon 38; *STK11* NM_000455.5: exons 1–9; *TERT* NM_198253.3: promoting regions (c.1–270G, c.1–43C), *TP53* NM_000546.6: exons 1–11; *TSHR* NM_000369.5: exon 10.

### Annex 2

For COBAS *EGFR* mutation test version 2 kit (Roche Diagnostic), the following *EGFR* mutations were detected: exon 18 (c.2156G>C, c.2155G>A, c.2155G>T), exon 19 (c.2240_2251del12, c.2239_2247del19, c.2238_2255del18, c.2235_2249del15, c.2236_2250del15, c.2239_2253del15, c.2239_2256del18, c.2237_2254del18, c.2240_2254del15, c.2240_2257del18, c.2239_2248delinsC, c.2239_2251delinsC, c.2237_2255delinsT, c.2235_2255delinsAAT, c.2237_2252delinsT, c.2239_2258delinsCA, c.2239_2256delinsCCA, c.2237_2253delinsTTGCT, c.2238_2252delinsGCA, c.2237_2248delinsGC, c.2237_2251del15, c.2236_2253del18, c.2235_2248delinsAATTC, c.2235_2252delinsAAT, c.2235_2251delinsAATTC, c.2253_2276del24, c.2237_2257delinsTCT, c.2238_2252del15, c.2233_2247del15), exon 20 (c.2303G>T, c.2369C>T (p.Thr790Met), c.2307_22308insGCCAGCGTG, c.2319_2320insCAC, c.2310_2311insGGT, c.2311_2312insGCGTGGACA, c.2309_2310delinsCCAGCGTGGAT), and exon 21 (c.2573T>G, c.2573_2574delinsGT, c.2582T>A.). The threshold of analytical sensitivity was 100 copies/mL of plasma.

## References

[1] Rolfo C., Mack P., Scagliotti G.V. (2021). Liquid biopsy for advanced NSCLC: A consensus statement from the International Association for the Study of Lung Cancer. J Thorac Oncol.

[2] Planchard D., Popat S., Kerr K. (2018). Metastatic non-small cell lung cancer: ESMO Clinical Practice Guidelines for diagnosis, treatment and follow-up. Ann Oncol.

[3] National Institutes of Health, National Cancer Institute Surveillance, Epidemiology, and End Results Program. Cancer stat facts: lung and bronchus cancer. https://seer.cancer.gov/statfacts/html/lungb.html.

[4] Leighl N.B., Page R.D., Raymond V.M. (2019). Clinical utility of comprehensive cell-free DNA analysis to identify genomic biomarkers in patients with newly diagnosed metastatic non-small cell lung cancer. Clin Cancer Res.

[5] Guisier F., Salaün M., Lachkar S. (2016). Molecular analysis of peripheral non-squamous non-small cell lung cancer sampled by radial EBUS. Respirology.

[6] Lachkar S., Perrot L., Gervereau D. (2022). Radial-EBUS and virtual bronchoscopy planner for peripheral lung cancer diagnosis: how it became the first-line endoscopic procedure. Thorac Cancer.

[7] Robin M., Mhanna L., Chaltiel L. (2021). Feasibility of comprehensive genotyping specimens from radial endobronchial ultrasonography and electromagnetic navigation bronchoscopy. ERJ Open Res.

[8] Wang Y., Liu Z., Yin H. (2018). Improved detection of EGFR mutations in the tumor cells enriched from the malignant pleural effusion of non-small cell lung cancer patient. Gene.

[9] Guo Z., Xie Z., Shi H. (2019). Malignant pleural effusion supernatant is an alternative liquid biopsy specimen for comprehensive mutational profiling. Thorac Cancer.

[10] Husain H., Melnikova V.O., Kosco K. (2017). Monitoring daily dynamics of early tumor response to targeted therapy by detecting circulating tumor DNA in urine. Clin Cancer Res.

[11] Goldman J.W., Karlovich C., Sequist L.V. (2018). EGFR genotyping of matched urine, plasma, and tumor tissue in patients with non-small-cell lung cancer treated with rociletinib, an EGFR tyrosine kinase inhibitor. JCO Precis Oncol.

[12] Jiang B.Y., Li Y.S., Guo W.B. (2017). Detection of driver and resistance mutations in leptomeningeal metastases of NSCLC by next-generation sequencing of cerebrospinal fluid circulating tumor cells. Clin Cancer Res.

[13] Ding S., Song X., Geng X. (2019). Saliva-derived cfDNA is applicable for EGFR mutation detection but not for quantitation analysis in non-small cell lung cancer. Thorac Cancer.

[14] Wang X., Ling L., Su H., Cheng J., Jin L. (2014). Aberrant methylation of genes in sputum samples as diagnostic biomarkers for non-small cell lung cancer: a meta-analysis. Asian Pac J Cancer Prev.

[15] Youssef O., Knuuttila A., Piirilä P., Böhling T., Sarhadi V., Knuutila S. (2018). Hotspot mutations detectable by next-generation sequencing in exhaled breath condensates from patients with lung cancer. Anticancer Res.

[16] Guibert N., Tsukada H., Hwang D.H. (2018). Liquid biopsy of fine-needle aspiration supernatant for lung cancer genotyping. Lung Cancer.

[17] Hannigan B., Ye W., Mehrotra M. (2019). Liquid biopsy assay for lung carcinoma using centrifuged supernatants from fine-needle aspiration specimens. Ann Oncol.

[18] Mack P.C., Banks K.C., Espenschied C.R. (2020). Spectrum of driver mutations and clinical impact of circulating tumor DNA analysis in non-small cell lung cancer: analysis of over 8000 cases. Cancer.

[19] Perrone M.E., Alvarez R., Vo T.T. (2021). Validating cell-free DNA from supernatant for molecular diagnostics on cytology specimens. Cancer Cytopathol.

[20] Finkelstein S.D., Bibbo M., Kowalski T.E. (2014). Mutational analysis of cytocentrifugation supernatant fluid from pancreatic solid mass lesions. Diagn Cytopathol Août.

[21] Roy-Chowdhuri S., Mehrotra M., Bolivar A.M. (2018). Salvaging the supernatant: next generation cytopathology for solid tumor mutation profiling. Mod Pathol.

[22] Pizzi M.P., Bartelli T.F., Pelosof A.G. (2019). Identification of DNA mutations in gastric washes from gastric adenocarcinoma patients: possible implications for liquid biopsies and patient follow-up. Int J Cancer.

[23] Sherwood J.L., Corcoran C., Brown H., Sharpe A.D., Musilova M., Kohlmann A. (2016). Optimised pre-analytical methods improve KRAS mutation detection in circulating tumour DNA (ctDNA) from patients with non-small cell lung cancer (NSCLC). PLoS One.

